# VITamin D supplementation in renAL transplant recipients (VITALE): a prospective, multicentre, double-blind, randomized trial of vitamin D estimating the benefit and safety of vitamin D_3_ treatment at a dose of 100,000 UI compared with a dose of 12,000 UI in renal transplant recipients: study protocol for a double-blind, randomized, controlled trial

**DOI:** 10.1186/1745-6215-15-430

**Published:** 2014-11-06

**Authors:** Marie Courbebaisse, Corinne Alberti, Sandra Colas, Dominique Prié, Jean-Claude Souberbielle, Jean-Marc Treluyer, Eric Thervet

**Affiliations:** Department of Physiology, Assistance Publique-hôpitaux de Paris, Hôpital Européen Georges Pompidou, F-75015 Paris, France; Univ Paris Descartes, Sorbonne Paris Cité, France; Departement of Epidemiology, Assistance Publique-hôpitaux de Paris, Hôpital Robert Debré, F-75019 Paris, France; Univ Paris Diderot, Sorbonne Paris Cité, UMR-S 1123, ECEVE, F-75019 Paris, France; Department of Clinical Research, Assistance Publique-hôpitaux de Paris, Hôpital Necker-Enfants Malades, Paris, France; Department of Physiology, Hôpital Necker-Enfants Malades, Assistance Publique-hôpitaux de Paris, Paris, France; Department of Nephrology, Hôpital Européen Georges Pompidou, Assistance Publique-hôpitaux de Paris, Paris, France

**Keywords:** Interventional trial, Vitamin D, Renal transplantation, Cancer, Cardiovascular events, Diabetes mellitus

## Abstract

**Background:**

In addition to their effects on bone health, high doses of cholecalciferol may have beneficial non-classic effects including the reduction of incidence of type 2 diabetes mellitus, cardiovascular disease, and cancer. These pleiotropic effects have been documented in observational and experimental studies or in small intervention trials. Vitamin D insufficiency is a frequent finding in renal transplant recipients (RTRs), and this population is at risk of the previously cited complications.

**Methods/design:**

The VITALE study is a prospective, multicentre, double-blind, randomized, controlled trial with two parallel groups that will include a total of 640 RTRs. RTRs with vitamin D insufficiency, defined as circulating 25-hydroxyvitamin D levels of less than 30 ng/ml (or 75 nmol/l), will be randomized between 12 and 48 months after transplantation to blinded groups to receive vitamin D_3_ (cholecalciferol) either at high or low dose (respectively, 100,000 UI or 12,000 UI every 2 weeks for 2 months then monthly for 22 months) with a follow-up of 2 years. The primary objective of the study is to evaluate the benefit/risk ratio of high-dose versus low-dose cholecalciferol on a composite endpoint consisting of *de novo* diabetes mellitus; major cardiovascular events; *de novo* cancer; and patient death. Secondary endpoints will include blood pressure (BP) control; echocardiography findings; the incidences of infection and acute rejection episodes; renal allograft function using estimated glomerular filtration rate; proteinuria; graft survival; bone mineral density; the incidence of fractures; and biological relevant parameters of mineral metabolism.

**Discussion:**

We previously reported that the intensive cholecalciferol treatment (100 000 IU every 2 weeks for 2 months) was safe in RTR. Using a pharmacokinetic approach, we showed that cholecalciferol 100,000 IU monthly should maintain serum 25-hydroxyvitamin D at above 30 ng/ml but below 80 ng/ml after renal transplantation. Taken together, these results are reassuring regarding the safety of the cholecalciferol doses that will be used in the VITALE study. Analysis of data collected during the VITALE study will demonstrate whether high or low-dose cholecalciferol is beneficial in RTRs with vitamin D insufficiency.

**Trial registration:**

ClinicalTrials.gov Identifier: NCT01431430.

**Electronic supplementary material:**

The online version of this article (doi:10.1186/1745-6215-15-430) contains supplementary material, which is available to authorized users.

## Background

### Vitamin D and vitamin D insufficiency in renal transplant recipients

Traditionally, vitamin D has been associated with bone health; its deficiency leads to rickets in children and osteomalacia in adults, and increases the risk of osteoporosis. More recently, vitamin D sufficiency has been associated with a reduced risk of many chronic diseases including type 2 diabetes mellitus (T2DM), cardiovascular diseases, cancers, and infectious diseases. All these diseases are more likely to occur in renal transplant recipients (RTR) than in the general population. Currently, the active form of vitamin D is currently used after kidney transplantation for the prevention of post-transplant bone loss [1] and the treatment of normocalcemic persistent secondary hyperparathyroidism [2–4]. Treatment with active vitamin D or its analogues will not compensate for inadequate 25-hydroxyvitamin D (25OHD), however. 25OHD is a substrate for 1α-hydroxylase (CYP27B1) in the kidney, and also in several extrarenal tissues, and these extrarenal tissues are dependent on adequate levels of 25OHD to ensure adequate local calcitriol production. Although there is no current consensus, vitamin D insufficiency is usually defined as 25OHD levels lower than 30 ng/ml (or 75 nmol/l) [5], because this limit is associated with a decrease in active intestinal calcium absorption [6] and with an increase in secretion of serum parathormone (PTH), which is involved in maintenance of normal serum calcium levels [5]. Furthermore, in interventional studies showing positive effects of vitamin D supplementation, the 25OHD levels reached in the treated groups were generally higher than 30 ng/ml [7].

The vitamin D insufficiency is present in more than 85% of adult RTRs [8]. Causes include: 1) insufficient vitamin D supplementation before and after transplantation; 2) increased 25OHD catabolism induced by immunosuppressive drugs [9] and by post-transplant persistent fibroblast growth factor-23 hypersecretion [10]; and 3) the reduced sun exposure recommended to RTRs to prevent skin cancers [11]. As a result, RTRs are now systematically advised to protect themselves from exposure to solar or artificial ultraviolet (UV) radiation. This represents a serious dilemma, as 80% to 90% of the human body’s requirements for vitamin D result from photosynthesis of the vitamin from 7-dehydrocholesterol in the skin by the action of UVB radiation. Therefore, careful monitoring of vitamin D status and oral supplementation to ensure that vitamin D insufficiency does not occur is of great importance for RTR.

### Supplementation of vitamin D after renal transplantation

Despite the high prevalence of vitamin D insufficiency in RTR, there is no general consensus regarding vitamin D supplementation after transplantation. In one study, [12], it was shown that high doses of vitamin D_3_ (100,000 IU cholecalciferol every other week for 2 months, equivalent to 6,600 IU/day) were able to correct 25OHD insufficiency in RTRs without significant side effects, and this regimen was also associated with a significant decrease in serum PTH concentration. However, this study also indicated that the dose of cholecalciferol used during the maintenance phase (100,000 IU every other month from months 6 to 12 post-transplantation) was insufficient to maintain serum 25OHD concentration above 30 ng/ml in half of patients [12]. Another study showed that 25,000 IU of cholecalciferol once a month failed to correct vitamin D insufficiency in RTRs, suggesting that a higher dose of cholecalciferol is necessary to maintain adequate 25OHD levels after transplantation [13]. The optimal dosage scheme was simulated from the data of a previous study [12] using a population pharmacokinetic approach. In order to maintain 25OHD concentrations between 30 and 80 ng/ml during the first year after renal transplantation, it was estimated that cholecalciferol dosing should be 100,000 IU once a month once correction of vitamin D insufficiency has been achieved [14].

### Anticancer properties of vitamin D

RTRs exhibit an increased incidence of cancers, especially of non-melanoma skin cancers and non-Hodgkin lymphoma. Data from the US Renal Data System indicate that at 3 years after renal transplantation, there is a 7.5% cumulative incidence of non-skin cancers and a 7.4% cumulative incidence of skin cancers in RTRs [15]. The role of vitamin D status in cancer risk has received strong experimental support from the consistent demonstration that activation of the vitamin D receptor (VDR) by locally produced calcitriol induces differentiation [16] and apoptosis [17], and inhibits cell proliferation [18] and angiogenesis [19]. Moreover, vitamin D and its metabolites stimulate mutual adherence of cells and intercellular communication through gap junctions, thereby decreasing metastatic potential and strengthening the inhibition of proliferation that results from tight intercellular physical contacts [20]. Many prospective case–control studies have shown that adults in the highest quantile of 25OHD levels have a decreased risk of colon [21] and breast [22] cancers compared with those in the lowest quantile. Furthermore, the risk of non-Hodgkin lymphoma is reduced by 30% to 40% in adults with high vitamin D intakes [23] or high levels of sun exposure [24]. Finally, retrospective studies suggest an association between low serum 25OHD level and death from cancer [25–27]. It must be mentioned that in a few observational studies a U-shaped (or rather an inverse J-shaped) curve between 25OHD serum levels and the risk of prostate [28], or pancreatic cancer [29] has been found (increased risk for both low and high serum 25OHD levels). However, it is a consensus that causality cannot be established from observational studies, so that randomized controlled trials (RCTs) evaluating the effect of vitamin D supplementation on the risk of cancer (versus placebo) are needed to firmly determine whether low and/or high vitamin D status increases the risk of cancer.

Such interventional studies of vitamin D supplementation in humans have yielded controversial data, however. In the Women’s Health Initiative (WHI) study, 36,282 women were randomized to receive either a placebo or 1000 mg calcium and 400 IU vitamin D_3_ daily. Although a strong negative relationship between baseline 25OHD levels and the incidence of colorectal cancer was found, no reduction in the incidence of cancers was observed in the treated group compared with the placebo group [30]. Of note, adherence to treatment was poor, and cholecalciferol dosage was considered to be too low by many experts in that study. Furthermore, women included in the WHI trial were allowed to continue to take the calcium/vitamin D supplementation that they were taking prior to enrolling in the study, so that some women in the placebo group received more vitamin D during the study than some women included in the calcium + vitamin D group. When the analysis was restricted to those women who were not taking calcium or vitamin D supplementation before the study, a significant reduction in the risk of total and invasive breast cancer, as well as in the risk of total cancer, was found [31]. A 4-year, double-blind, randomized, placebo-controlled trial comprising 1,180 postmenopausal women showed a significant decreased risk of cancers in the group receiving calcium (1,500 mg/day) plus vitamin D_3_ (1,100 IU vitamin D3/day) supplementation [32], whereas another 3-year, double-blind, randomized, placebo-controlled trial comprising 5,292 patients aged over 70 years showed no reduction in cancer incidence with vitamin D_3_ 800 IU per day [33]. To our knowledge, no interventional study has reported that vitamin D supplementation increases the risk of any cancer, although it must be emphasized that the doses used were generally too low and the duration of the studies too short to conclude this definitely.

The potential protective role of vitamin D against cancer risk was also assessed in RTRs [34]. In a cohort of 363 RTRs followed up during 3 to 5 years after transplantation, a higher incidence of post-transplant cancers was observed in patients with pre-transplant 25OHD concentrations less than 10 ng/ml (13.7% vs. 3.7% for those with 25OHD levels >30 ng/ml, *P* =0.007). Another study reported no association between cancer incidence and vitamin D status over a 10-year follow-up period after renal transplantation [35], although a single repletion study showed a decrease in cancer risk in RTRs treated with active vitamin D [36]. Whether these results can be explained by risk segregation with cancer type, particularly viral-related cancers, remains to be established.

The relationship between sun exposure and non-melanoma skin cancer also remains to be elucidated. Even though the role of sun exposure has been demonstrated, it should be note that VDR knockout (KO) mice develop UVB-induced skin cancers more rapidly and more frequently than wild-type mice, suggesting a potential protective role for 25OHD against non-melanoma skin cancers [37]. In RTR, regular application of sun protection factor (SPF) 50 sunscreen is associated with fewer skin lesions but also with lower 25OHD levels [38]. The association of higher levels of 25OHD with an increased risk of skin cancer can be explained by greater UV exposure [39]. These data highlight the difficulties of drawing conclusions using only epidemiological and not interventional studies.

### Anti-diabetic properties of vitamin D

According to the diagnostic criteria and post-transplantation delay, *de novo* T2DM occurs in 10% to 30% of RTR, mainly due to corticosteroid and tacrolimus treatment [40]. The potential effects of vitamin D on insulin secretion and insulin resistance are supported by experimental data [41]. First, VDR [42] and CYP27B1 [43] are expressed in pancreatic β cells, and vitamin D responsive elements (VDRE) have been identified in the promoter of the human gene encoding insulin [44]. Second, *in vitro* studies have shown that calcitriol stimulates transcription of the insulin gene, expression of insulin receptor, and glucose transport [41]. Third, VDR knockout (KO) mice have abnormal insulin secretion [45], and vitamin D_3_ supplementation increases glucose tolerance and insulin secretion in vitamin D-deficient rats [46]. In humans, serum 25OHD concentrations are inversely correlated with T2DM prevalence [41, 47–49]. Vitamin D insufficiency is also associated with increased glycated hemoglobin (HbA1c) levels [50] and resistance to insulin [47]. The influence of vitamin D on the resistance to insulin may be partly mediated by calcitriol, through control of the gene encoding adiponectin [51]. In a very recent paper, it was reported that vitamin D and calcium supplementation decreased serum interleukin (IL)-6 and tumour necrosis factor-α concentrations in patients with T2DM, and might thus improve systemic inflammation in this disease [52]. A recent meta-analysis of RCTs showed that active or native vitamin D supplementation improved fasting glycaemia and insulin resistance in patients with glucose intolerance, but had no effect on HbA1c levels [53]. However, another recent meta-analysis found no effects of vitamin D supplementation on glucose homeostasis or diabetes prevention, although a trend (*P* =0.06) towards reduction in fasting blood glucose in patients with pre-diabetes, and a significant reduction in homeostatic model assessment of insulin resistance (HOMA-IR) after exclusion of the studies that administered a single large dose of vitamin D were reported [54]. These authors indicated also that definitive conclusions may be limited because of heterogeneity in the studies, variable risk of bias, and the short-term follow-up duration of the available evidence to date, again emphasizing, as mentioned above for the potential effects of vitamin D on cancers, the need for large, well-conducted RCTs. To date, no study has reported the potential effect of active or native vitamin D on *de novo* T2DM after transplantation.

### Effects of vitamin D on the cardiovascular system

In comparison with the general population, RTRs have an increased cardiovascular risk secondary to both traditional and non-traditional risk factors (50-fold higher in RTRs) [55]. Observational and experimental data argue in favour of a potential protective role of vitamin D against cardiovascular disease. Cross-sectional studies have indicated that vitamin D deficiency is associated with arteriosclerosis and endothelial dysfunction in patients with end-stage renal disease [56]. Several prospective case–control studies have reported a strong association between low circulating levels of 25OHD and an increased risk of major cardiovascular events such as myocardial infarction, stroke, congestive heart failure [57, 58], and cardiovascular disease death [59–62]. These associations remained significant after adjustment for other risk factors for cardiovascular disease.

Possible explanations for these findings involve both direct and indirect effects of vitamin D on cardiovascular function. Direct effects are supported by the fact that cardiomyocytes, vascular smooth muscle cells, and endothelial cells express both VDR and the CYP27B1 enzyme [63]. Furthermore, genes upregulated during myocardial hypertrophy (such as atrial natriuretic peptide) possess VDREs, and are suppressed by calcitriol in animal and cell models [64]. Similarly, in cultured cells, calcitriol inhibits cardiomyocyte proliferation [65], and stimulates vascular smooth muscle cell proliferation and vascular endothelial growth factor expression by these cells [66]. Calcitriol also modulates contractile performances of isolated rat and mouse cardiomyocytes [67, 68].

Potential indirect consequences of vitamin D deficiency may underlie its role as a risk factor for cardiovascular dysfunction. VDR but also CYP27B1 KO mice have high BP levels and cardiac hypertrophy due to increased activation of the renin-angiotensin system (RAS) [69], and calcitriol treatment inhibits renin activation and decreases BP and cardiac hypertrophy in CYP27B1 KO mice [70]. Calcitriol also reduces the expression of the metalloproteinases (MMP) MMP2 and MMP9 [71]; expression of these two MMPs appears to promote vascular calcification [72]. In a recently published mendelian randomization study performed in 142,255 individuals, increase in an allele score based on variants of genes that affect 25OHD synthesis or substrate availability, and used as a proxy for 25OHD concentration, was found to be significantly associated with reduced odds of hypertension [73]. Even though no controlled interventional trials with clinical cardiovascular endpoints have been performed, numerous studies have shown either no or positive effects of vitamin D supplementation on intermediary parameters potentially related to cardiovascular health such as BP, endothelial or left ventricular function, and lipid profile [74]. Interestingly, a meta-analysis of 11 randomized trials either with active or native vitamin D confirmed this reduction in systolic BP in patients with hypertension, and suggested that native vitamin D produced a greater fall in systolic BP than activated compounds [75]. In that meta-analysis, positive effects of vitamin D supplementation on BP were modest and limited to patients with hypertension. Another potential cause explaining negative results of vitamin D trials on BP was suggested by the results of a recent 20-week study performed in 130 patients with moderate hypertension who received either 3,000 IU vitamin D_3_ daily or a placebo [76]. The primary objective, ambulatory BP (24 h BP), was not significantly modified in the vitamin D group compared with placebo in the intent-to-treat (ITT) analysis, although central BP, a secondary endpoint, was significantly reduced. However, in a *post hoc* analysis limited to the 92 patients with a baseline 25OHD level of less than 32 ng/ml, 24 h BP was modestly but significantly reduced. This may partly explain why ITT meta-analyses of studies on the effect of vitamin D on BP that include both patients with normal and patient with high BP, as well as patients with or without vitamin D deficiency, may appear negative, while the effect seems to be positive in patients with hypertension and vitamin D insufficiency.

Interventional studies targeted to the effect of vitamin D supplementation on major cardiovascular events and using death as a primary endpoint do not yet exist. In a secondary analysis of the RECORD trial on four pre-specified outcomes, vitamin D supplementation was found to protect against cardiac failure (HR =0.75; CI 0.58 to 0.97), but not against myocardial infarction or stroke [77]. However, several recently published meta-analyses of interventional studies (whose primary endpoint was not cardiovascular events) found no (positive or negative) effect of vitamin D supplementation on major cardiovascular events [77–79], although a slight, but significant decrease in all-cause mortality was reported [79–81]. To date, the few, weakly powered, observational studies performed in RTRs have found no robust association between serum 25OHD levels and cardiovascular risk factors [35, 82, 83]. The apparent discrepancy between the strong association of vitamin D deficiency with major cardiovascular events and the lack of strong evidence of a reduction in cardiovascular risk with vitamin D supplementation may be due to reverse causation, which frequently affects observational studies (low vitamin D being the consequence rather than the cause of the disease under study).

However, because vitamin D intoxication in humans may lead to vascular calcifications, the benefit/risk ratio of supraphysiologic dosages of vitamin D should be evaluated, especially in RTRs having an increased cardiovascular risk. Indeed, a biphasic effect of vitamin D on the risk of vascular calcifications is likely [84]. It has been recently reported from observational studies that the risk of all-cause mortality [85] and the risk of major cardiovascular events [86, 87] increased if serum 25OHD levels were below 20 ng/ml but also if levels were above 40 ng/ml. Although no causality can be concluded from observational studies, these results should incline the medical community to be cautious with regard to high-dose vitamin D supplementation and cardiovascular health.

## Methods

### Objectives

The VITALE study is designed to evaluate whether high doses of cholecalciferol in RTRs with vitamin D insufficiency has beneficial effects upon the late post-transplant outcome compared with low-dose supplementation. As a primary outcome, we will evaluate the impact of cholecalciferol on a composite endpoint including *de novo* DM (fasting glycaemia >7 mmol/l or glycaemia >11 mmol/l), major cardiovascular events (acute coronary heart disease, acute heart failure, lower-extremity arterial disease, cerebrovascular disease), *de novo* cancer, and patient death. Secondary objectives will compare the effects of high-dose versus low-dose cholecalciferol on BP and on BP control (number and dosage of antihypertensive drugs), echocardiography findings, infections (including cytomegalovirus, *Pneumocystis*, nocardial infection, cryptococcal infection, aspergillosis), acute rejection episodes, renal allograft function including Modification of the Diet in Renal Disease (MDRD)-v4 estimated glomerular filtration rate (eGFR) [88], proteinuria and graft survival, and mineral metabolism biologically (serum calcium, phosphate, 25OHD and PTH) and clinically (graft nephrolithiasis, bone mineral density, measured body weight, and incidence of fractures) relevant parameters.

We have noted that, according to the published literature, some vitamin D experts especially involved in the cardiovascular field consider that optimal 25OHD concentration for cardiovascular health probably lies within a narrow range of 30 to 40 ng/ml [89, 90]. We took this advice into account, and one of the main objectives of our VITALE trial is thus to evaluate whether our treatment scheme, which probably will increase the 25OHD concentration above 40 ng/ml in a significant percentage of patients, is safe, especially in terms of cardiovascular health.

### Study population and sample size

The study sample will consist of 640 RTRs found to be lacking sufficient vitamin D (25OHD <30 ng/ml) 12 to 48 months post-transplantation with 320 subjects in each study group. Inclusion and exclusion criteria are detailed in the Appendix. The sample size calculation was performed according to the following assumptions: The principal criteria will be the occurrence of one of the events of the composite endpoint including *de novo* DM, major cardiovascular events, *de novo* cancer, and patient death. Over a 2-year follow-up period, we estimate, according to previous reports in the literature, that the incidence of a first event of the composite endpoint will be around 22% in our population (6% for *de novo* T2DM [91, 92], 6% for major cardiovascular events [93–95], 7% for *de novo* cancers [15, 96], and 3% for patient death). This 22% global estimated incidence does not take into account the fact that some patients may experience several events of the composite endpoint during the follow-up period. According to the results of the numerous observational studies showing an inverse association between 25OHD concentrations and various clinical events and to the results of the few interventional trials showing effects of high-dose vitamin D supplementation on these events (detailed above),we estimate that the overall decrease in the incidence risk of a first event of the composite endpoint will be around 40% in the high-dose group compared with the low-dose group (33% decrease for *de novo* DM, 40% decrease for major cardiovascular events and 50% decrease for *de novo* cancer). Thus, to demonstrate the reduction in the incidence of a first event of the composite endpoint from 22% in the low-dose group to 13% in the high-dose group, with a 90% power and 5% α risk error, 582 RTRs (291 in each group) will be required. Taking into account that 10% of patients will not be evaluable, a total of 640 RTRs will be required. The inclusion of a total of 480 RTRs would allow us to test the same hypothesis with a power of 80%.

### Study design and setting

**Figure 1:**
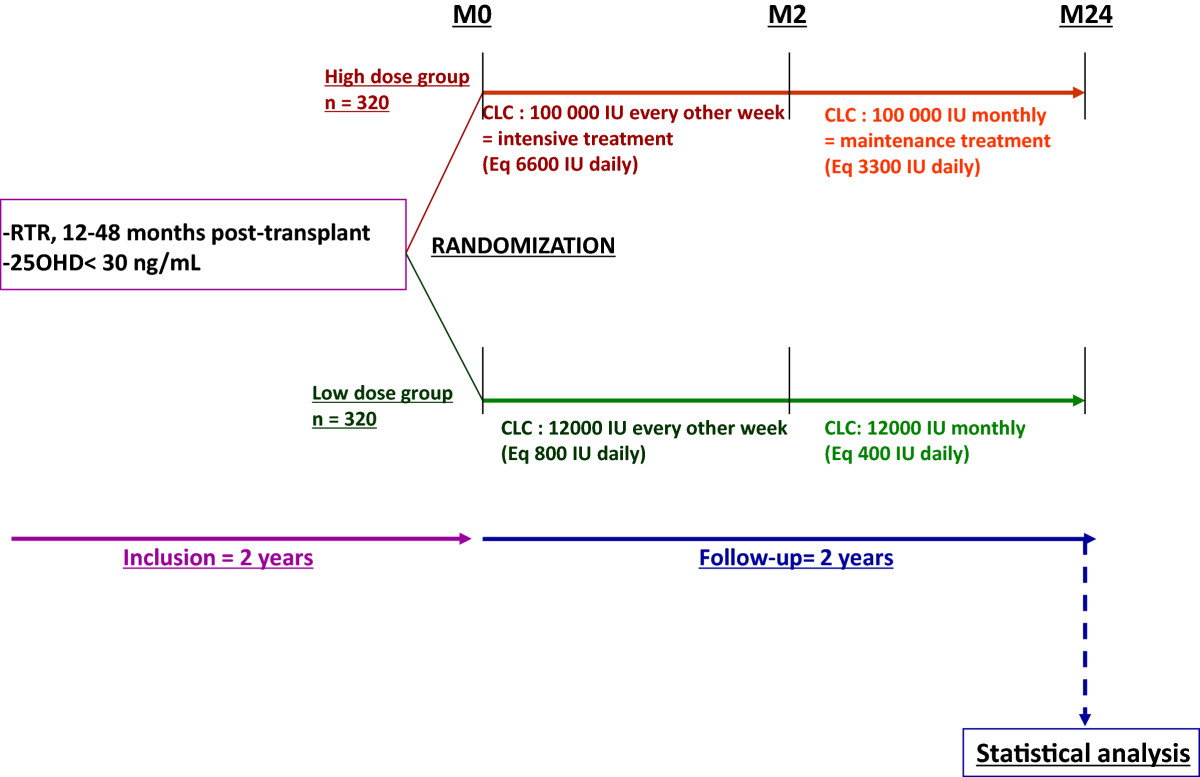
**Flowchart of the VITALE study.** RTR with 25OHD insufficiency (25OHD <30 ng/ml) will be included 12 to 48 months after renal transplantation in 30 transplantation departments in France, and randomized to receive either high-dose cholecalciferol treatment (100,000 IU every other week, equivalent to 6,600 IU daily for 2 months, then 100,000 IU monthly, equivalent to 3,300 IU daily for 22 months) or low-dose cholecalciferol treatment (12,000 IU every other week, equivalent to 800 IU daily for 2 months, then 12,000 IU monthly, equivalent to 400 IU daily for 22 months, which is the French recommended dietary intake). Duration of patient follow-up will be 2 years. VITALE is a double-blind study as a single-dose vial of 100,000 or 12,000 IU have exactly the same appearance. We aim to include 320 RTR sin each group (high-dose group and low-dose group) over a period of approximately 2 years. Statistical analysis will be performed at the end of the study.

The VITALE study is a prospective, multicentre, double-blind, randomized trial with two parallel groups. Patient recruitment, kidney transplants, postoperative care, and follow-up are currently conducted in 30 transplantation departments in France, and there are about 2,700 kidney transplantations performed each year in France. In the absence of vitamin D supplementation, approximately 80% of RTRs are expected to have insufficient vitamin D. Of these patients, 20% to 30% are expected to fulfil the inclusion criteria and to agree to participate in the study. Consequently, the recruitment phase will last approximately 2 years, with a follow-up period of 2 years for each subject. The flowchart of the VITALE study is shown in Figure 1.

### Randomization and blinding

If all inclusion and none of the exclusion criteria are met and informed consent has been obtained, each RTR will be included in the study and allocated a randomization number used for assignment to one of the two treatment arms. The randomization list will be computer-generated and created using nQuery Advisor software (v6.01). The randomization will be stratified by centres, and blocks of four will be used in order to facilitate treatment distribution by the central pharmacy. The size of the blocks will be unknown to the investigators. The two treatment regimens will be randomly allocated in a 1:1 ratio. The attribution arm will be given centrally by Cleanweb® software after the validation of inclusion criteria, and this software also allows entering trial data for each patient. Participants, investigators, and outcome assessors will be blinded to the allocated treatment. Blinding will be ensured by the use of investigational products that are identical in packaging, labelling, appearance, smell, and taste. Unblinding will occur only in cases of emergency or at the conclusion of the study. In most cases, discontinuation of the treatment should be sufficient without the need for unblinding. Access to the randomization list will be restricted to the pharmacist involved in the study and a research assistant.

### Study intervention

At 12 to 48 months after renal transplantation, RTRs found to have 25OHD levels below 30 ng/ml and who fulfil all of the inclusion and none of the exclusion criteria will be randomized to receive either high-dose y (100,000 UI every 2 weeks for 2 months, then monthly for 22 months) or low-dose (12,000 UI every 2 weeks for 2 months, then monthly for 22 months) oral cholecalciferol therapy. With a total follow-up of 2 years. Each 2 ml vial will contain either cholecalciferol 100,000 IU or 12,000 IU plus butylhydroxytoluene 0.2 mg, saccharin 1.2 mg, sorbic acid 4.00 mg, lemon essential oil 6.0 mg, with glycosylated polyoxyethylenated glycerides making the quantity up to 2 ml. High-dose and low-dose vials will be produced by Crinex (Montrouge, France). It will be recommended that cholecalciferol be ingested concomitantly with a fatty meal to improve intestinal absorption of vitamin D_3_ [97]. The intensive phase of the high-dose arm was previously demonstrated to correct 25OHD insufficiency in RTRs [12], and the maintenance phase was demonstrated in a theoretical scheme to maintain 25OHD above 30 ng/ml after kidney transplantation [14]. The 12,000 IU cholecalciferol monthly dose during the 22 month-maintenance phase, equivalent to 400 IU daily, was chosen because it is in agreement with the French recommended dietary intake [98], and this dose has been proven to avoid severe vitamin D deficiency and osteomalacia [99]. To evaluate adherence to treatment, patients will be asked to return the treatment boxes containing the empty ampoules at each follow-up visit.

The two side effects caused by vitamin D_3_ that may occur are increase in serum calcium levels or in urinary calcium excretion. At serum calcium levels >2.85 mmol/l, serum phosphate levels >1.8 mmol/l or in cases of major increase in urinary calcium excretion (increase in fasting baseline urinary calcium/creatinine ratio ≥0.4 mmol/mmol in the absence of furosemide introduction), the aforementioned monthly vitamin D_3_ administration will be discontinued. If the biological abnormalities cited above persist after vitamin D_3_ re-introduction, cholecalciferol treatment will be halted. Cholecalciferol treatment will be immediately discontinued in cases of proven graft nephrolithiasis (except for uric acid calculi, whose composition has been proven by calculi analysis) or nephrocalcinosis, symptomatic hypercalcemia, or pregnancy occurrence. VITALE is an ITT study. Consequently, in cases of cholecalciferol discontinuation, patient follow-up will be maintained according to the study guidelines until the end of the 24 month follow-up period.

### Concomitant medication

Most RTRs are treated with an immunosuppressive therapy consisting of mycophenolate mofetil or sodicum mycophenolate, tacrolimus, or ciclosporin, with (in most cases) or without prednisone. Steroid boli, monoclonal antibodies, and polyclonal antibodies are administered to treat acute rejection episodes. Medications will be allowed during the study, with the exception of active or native vitamin D_2_, active or native D_3_, and multivitamin complexes likely to contain vitamin D. Drugs with anti-secretory and gastric-dressing properties will be allowed, but will have to be ingested at least 4 hours after cholecalciferol treatment.

### Study procedure

#### Inclusion visit

At 12 to 48 months after kidney transplantation, each RTR found to have 25OHD levels below 30 ng/ml during a routine visit will be given information about the VITALE study, its purpose, putative benefits, and possible risks, and will be invited to participate in the trial. All patients will be informed that the participation in that study is voluntary, and that they may refuse to participate or withdraw from the trial at any time without giving reasons and without loss of benefits. RTRs who do not agree to participate in the study will receive standard care. If the RTR agrees, they will sign a written informed consent form, and demographic, clinical, and biological baseline data will be collected. In addition, daily calcium intake will be evaluated by means of a questionnaire.

#### Randomization

During the week following the inclusion visit, RTRs who agree to participate in the study will be randomized to receive either oral high-dose vitamin D_3_ therapy or oral low-dose vitamin D_3_ therapy. The day of the randomization defines the first day of the study.

#### Patient follow-up

Five visits will be performed after randomization (Table 1). Vitamin D measurements after randomization will be centralized and performed at the end of the study. Given the absence of standardization and a reference method of vitamin D measurement when we submitted the VITALE trial to funders (early 2011), we planned to use the most referenced method (radioimmunoassay; Diasorin, Stillwater, MN, USA) [100]. Since then, a reference method has been accepted [101] and is now being used in an international program, the Vitamin D Standardization Program (VDSP), to harmonize vitamin D results [102]. We thus will use the assay (either immunoassay or commercial LC-MS-MS assay) that at the end of the VITALE study will provide results that are closest to the reference method [103]. This will allow us to determine with the best possible precision the optimal threshold of 25OHD level to prevent the occurrence of the clinical events constituting the principal criteria. In order to assess compliance, patients will be asked to return their empty vials at each follow-up visit.

**Table 1:** **Study procedures**

Date	Patients selection	Inclusion visit		M1	M2	M3	M6	M12	M18	M24
Cholecalciferol (high versus low dose)			**R = T0** ^**1**^	**Intensive phase (2 months)**		**Maintenance phase (22 months)**				
Written informed consent/checking of the inclusion and exclusion criteria		X								
Medical history entry		X								
Clinical examination, blood pressure, body weight and height		X				X	X	X	X	X
Current treatment		X				X	X	X	X	X
Notification of events constituting the primary composite criteria and the secondary criteria						X	X	X	X	X
Checking of treatment compliance						X	X	X	X	X
Notification of tolerance and side effects^2^						X	X	X	X	X
β-human chorionic gonadotropin if relevant		X								
25OHD	< 30 ng/ml	X				X^1^		X^1^		X^1^
Serum calcium^2^	<2.7 mmol/l	X				X	X	X	X	X
Serum phosphate^2^	<1.5 mmol/l	X				X	X	X	X	X
Fasting urinary calcium^2^		X				X	X	X	X	X
Fasting urinary creatinine^2^		X				X	X	X	X	X
Serum creatinine	<250 μmol/l	X				X	X	X	X	X
MDRD estimated glomerular filtration rate		X				X	X	X	X	X
Urinary protein/creatinine ratio		X						X		X
Urinary microalbumin/creatinine ratio		X						X		X
Fasting glycaemia	< 7 mmol/l	X				X	X	X	X	X
HbA1C		X						X		X
Liver function tests		X								X
Lipid profile		X						X		X
Complete blood count		X						X		X
Serum parathyroid hormone		X						X		X
Bone mineral density		X								X
Echocardiography^2^		X						X		X
DNA collection^3^		X								
Serum collection^3^		X				X		X		X

Table legend: M1 (M2, 3…): month one (two, three…) after randomization; MDRD: Modification of the Diet in Renal Disease; R: randomization; T0: time zero corresponding to the randomization of the patient.

^1^R: randomization represents the time 0 (T0). Intensive phase: cholecalciferol 100,000 IU or 12,000 IU every two weeks for 2 months. Maintenance phase: cholecalciferol 100,000 IU or 12,000 IU monthly for 22 months.

^2^Safety data. Echocardiography results will have to notify valvular calcifications.

^3^Samples for which the dosage (25OHD) and/or conservation will be centralized.

### Statistical analysis

#### Descriptive analysis

Patients will be described according to their initial treatment group attributed after the randomization. A flowchart of enrolled and analysed patients will be provided. Demographic, clinical, and biological characteristics recorded at time of randomization will be described. Descriptive analyses will use numbers (percentages) for qualitative variables, mean ± standard deviation or median (first to third quartiles) as appropriate for quantitative variables, and Kaplan-Meir curves or cumulative incidences in the presence of competing risks for time to event. No statistical test will be formally performed to compare the initial characteristics of randomized groups.

#### Statistical analysis

The ITT analysis will involve data on all patients in each dose group. All patients who are lost to follow-up for any reason will be treated as failures at the time of last contact or at the time of an event that results in discontinuation, whichever occurs first. The per-protocol set will exclude from the full analysis the set non-compliant patients, patients who received less than 50% of the treatment, and patients who received concomitant administration of an excluded treatment.

All events occurring during the 2 years of follow-up will be recorded. The principal criteria (occurrence of the first event constituting the composite endpoint) will be compared between the two groups using the Cox model that takes into account the stratification by the centre. Secondary criteria will be compared using tests corresponding to the nature of the variable: *χ*^2^ or Fisher’s exact test for qualitative variables, parametric or non-parametric variance analysis for quantitative variables, and log-rank or Gray’s test for time to event. The secondary parameters that will be analysed are the following: the incidence of each of the events constituting the composite primary endpoint; BP measurement, and number, drug classes and doses of antihypertensive drugs necessary to reach normal BP; preventive coronary revascularisation; evolution of the left ventricular ejection fractions and of the left ventricular wall thickness assessed by transthoracic echocardiography; vitamin D insufficiency correction; evolution of the fasting urinary calcium/creatinine ratio and of serum calcium, phosphate, and PTH concentrations; bone fracture incidence; changes in body height; changes in bone mineral density at the lumbar spine and femoral neck; incidence of infectious episodes and type of infection; incidence of treated acute rejection episodes; evolution of MDRD eGFR, of urinary protein/creatinine ratio, and of urinary microalbuminuria/creatinine ratio; graft survival; evolution of HbA1C in the absence of DM; evolution of HbA1C and of anti-diabetic treatments in cases of *de novo* DM; or incidence of cholecalciferol-related side effects (hypercalcaemia, calcic nephrolithiasis and other spontaneously reported side effects). Adjustment for multiple testing will be performed using the Hochberg procedure for the components of the primary criteria. We will also describe in detail each adverse event (AE) that occurs during the study, and we will report the overall rate of occurrence of AE, the rate of withdrawal from the study due to an AE, the overall rate of patients with at least one clinically significant laboratory abnormality, the rate of AEs attributable to treatment (possible or probable relationship), and the rate of serious AEs. The percentage of patients with each of the AEs associated with cholecalciferol and the intensity of these AEs will be specified.

Some additional p*ost hoc* subgroup analyses will also be performed (according to, for example, baseline 25OHD levels, achieved 25OHD levels, VDR and vitamin D binding protein polymorphisms, and corticosteroid treatment, among others).

### Approval of the ethics committee and the regulatory authority

The proposed study will be conducted in accordance with the Declaration of Helsinki and with French law, and will subscribe to the principles outlined in the International Conference on Harmonization on Good Clinical Practice, 2002. A favourable opinion was delivered by the Ethics CommitteeCCP-IDF1 (Committee for the Protection of Persons-Ile-de France 1) with the reference number 2011-mars-12559. In France, every trial is approved by a single ethics committee for each involved centre, according to the legislation for the studies funded by a research grant from the French Ministry of Health, as is the case for our study (VITALE = PHRC P100103). The local ethics committee of each centre is not subsequently involved. Furthermore, the study has been registered in a public clinical trial database (ClinicalTrials.gov Identifier: NCT01431430).

## Discussion

### Risk analysis

Vitamin D intoxication does not occur if 25OHD concentration remains at less than 150 ng/ml [104]. There is no evidence that the margin of safety for vitamin D_3_ differs in patients with renal disease compared with the rest of the population [104]. Several studies have shown that daily doses of vitamin D significantly higher than the recommended dietary intake (more than 4,000 IU per day and up to 10,000 IU per day) do not affect urinary calcium or serum calcium levels [105–108]. There have also been studies of the effect of spaced high doses, which likewise reported no indication of vitamin D toxicity [107, 109]. Most importantly, we have previously reported that the high-dose intensive cholecalciferol treatment we intend to use (100,000 IU every 2 weeks for 2 months) was safe in RTRs [12]. Using a pharmacokinetic approach, we determined that cholecalciferol 100,000 IU monthly should maintain 25OHD above 30 ng/ml but below 80 ng/ml [14]. Taken together, these results suggest that the doses of cholecalciferol to be used in the VITALE study will be safe. No other AEs of vitamin D supplementation have been reported in the published intervention studies. However, as several observational studies have reported an inverse J-shaped curve between 25OHD serum levels and several hard endpoints such as cardiovascular major events and some cancers, we will be monitoring carefully for any signs of possible AEs.

For our study, we have chosen a composite criterion for efficacy. The main advantages supporting this choice are that it increases statistical efficiency because of a higher event rate, which reduces sample size requirement. In addition, it helps investigators avoid an arbitrary choice between several important outcomes that refer to the same disease process. Composite outcomes can be misleading when treatment effects vary across components with very different clinical importance. In our study, the rate of each event except for death is of similar magnitude. Moreover, we hypothesize that the rate reduction of each event composing the endpoint will be between 30% and 50%, giving a mean reduction of 40% overall. We also hypothesize that the drug will not affect death, but death is being included in the composite endpoint because it is a competing risk with major cardiovascular events. In order to limit misleading interpretation, we will clearly present data for all components and discuss the role of each in the total reduction of the composite event rate.

### Other interventional studies aiming at studying the effects of vitamin D_3_ on extra-osseous criteria after renal transplantation

Two other studies are also testing the effect of native vitamin D on extra-osseous diseases after renal transplantation [110]. The VITA-D study (Vitamin D_3_ Substitution in Vitamin D Deficient Kidney Transplant Recipients; ClinicalTrials.gov Identifier: NCT00752401) is also a double-blind, randomized, placebo-controlled study of RTRs deficient in vitamin D, focusing on the impact of cholecalciferol substitution on graft function (MDRD eGFR), incidence of acute rejection episodes, and post-transplant infections within the first year after transplantation. In total, 200 RTRs with 25OHD of less than 20 ng/ml at time of transplantation will be randomized to receive either cholecalciferol (6,800 IU/day during one year) or placebo [111]. The CANDLE-KIT study (Correcting Anemia and Native Vitamin D Supplementation in Kidney Transplant Recipients; ClinicalTrials.gov Identifier: NCT01817699) is another open-label RCT with four arms: 1) no intervention: low haemoglobin (Hb) target (Hb level: ≥9.5 and <10.5 g/dL) without cholecalciferol; 2) low Hb target with cholecalciferol 1,000 IU/day; 3) high Hb target (Hb level: ≥12.5 and <13.5 g/dL) without cholecalciferol, and 4) the experimental arm: high Hb target with cholecalciferol 1,000 IU/day. This study will recruit 324 RTRs, who are at least q year post-transplantation. The primary endpoint will be the change in allograft kidney function using MDRD eGFR. Among he secondary endpoints are urinary markers of kidney injury, the dose of methoxypolyethylene glycol epoetin β required to maintain the target haemoglobin level, BP, cardiac biomarkers, left ventricular mass index, acute cellular rejection, bone-turnover markers, intact PTH, bone mineral density, cardiovascular events, all-cause death, and cancer development or recurrence. Numerous criteria of these two studies are part of the primary composite endpoint or of the secondary endpoints in the VITALE study.

### Conclusion and perspectives

In addition to its classic effects on bone and mineral metabolism, vitamin D displays a wide spectrum of potential non-classic effects that are especially relevant for the care of RTRs. These pleiotropic effects have been documented in observational and experimental studies or small intervention trials, which most often evaluated intermediate parameters. The time has now come for large placebo-controlled trials in RTRs, using larger dosages of vitamin D than the current recommended intakes, and targeting clinical endpoints. The VITALE study has been designed to demonstrate that high doses of vitamin D can reduce the risk of extra-osseous diseases without inducing AEs, and also aims to determine the necessary levels of 25OHD to achieve these effects. Furthermore, the RTR population may give interesting clues to the effects of vitamin D in the general population, as cardiovascular, diabetes mellitus, and cancer complications are much more frequent in the former than in the latter.

## Trial status

Patient recruitment ongoing.

## Appendix

### Eligibility criteria

#### Inclusion criteria

-  RTRs who are between 12 and 48 months after transplantation with stable renal function during the past 3 months
-  Vitamin D insufficiency, defined as a concentration of 25OHD lower than 30 ng/ml
-  Aged between 18 and 75 years old
-  Capable of understanding the advantages and the risks of the study
-  Have social security health insurance
-  Have provided written informed consent

#### Exclusion criteria

-  Calcaemia >27 mmol/l
-  Phosphataemia >15 mmol/l
-  Serum creatinine >250 μmol/l
-  Receiving treatment with an active form of vitamin D,which cannot be interrupted
-  Transplant of an organ other than the kidney
-  Type 1 or type 2 DM
-  Medical history of granulomatosis
-  Primary hyperoxaluria
-  Proven malabsorption of liposoluble vitamins
-  Simultaneous participation in another therapeutic clinical trial
-  Drug addiction or a psychiatric disorder
-  Pregnancy or breast-feeding
-  Vitamin D hypersensitivity

## Authors’ information

MC, MD, PhD, Department of Physiology, Hôpital Européen Georges Pompidou, Assistance Publique-hôpitaux de Paris, Paris, France and Paris Descartes University: scientific supervisor of the study and corresponding author.

CA, MD, PhD, Departement of Epidemiology, Hôpital Robert Debré, Assistance Publique-hôpitaux de Paris, Paris, France: responsible for statistical analysis.

SC, PhD, Department of ClinicalResearch, Hôpital Necker-Enfants Malades, Assistance Publique-hôpitaux de Paris, Paris, France: project coordinator.

DP, MD, PhD, Department of Physiology, Hôpital Necker-Enfants Malades, Assistance Publique-hôpitaux de Paris, Paris, France and Paris Descartes University: responsible for 25-hydroxy vitamin D bioassay.

JCS, PhD, Department of Physiology, Hôpital Necker-Enfants Malades, Assistance Publique-hôpitaux de Paris, Paris, France: responsible for 25-hydroxy vitamin D bioassay.

JMT, MD, PhD, Department of Clinical Research, Hôpital Necker-Enfants Malades, Assistance Publique-hôpitaux de Paris, Paris, France and Paris Descartes University: responsible for the clinical research unit managing the study.

ET, MD, PhD, Department of Nephrology, Hôpital Européen Georges Pompidou, Assistance Publique-hôpitaux de Paris, Paris, France and Paris Descartes University: principal investigator of the study.

All authors read and approved the final manuscript.

## Authors’ original submitted files for images

Below are the links to the authors’ original submitted files for images. Authors’ original file for figure 1

## Abbreviations

DM: Diabetes mellitus
eGFR: Estimated glomerular filtration rate
Hb: Haemoglobin
CYP27B1: 1α-hydroxylase
25OHD: 25-hydroxyvitamin D
KO: Knockout
MMP: Metalloproteinase
PTH: Parathormone
RAS: Renin-angiotensinsystem
RTRs: Renal transplant recipients
VDR: Vitamin D receptor
VDRE: Vitamin D responsive elements.
