# Supplementary figures and images for: VITamin D supplementation in renAL transplant recipients (VITALE): a prospective, multicentre, double-blind, randomized trial of vitamin D estimating the benefit and safety of vitamin D3 treatment at a dose of 100,000 UI compared with a dose of 12,000 UI in renal transplant recipients: study protocol for a double-blind, randomized, controlled trial
Source: Trials. 2014 Nov 6;15:430. doi: 10.1186/1745-6215-15-430 (PMC4233037; doi:10.1186/1745-6215-15-430)

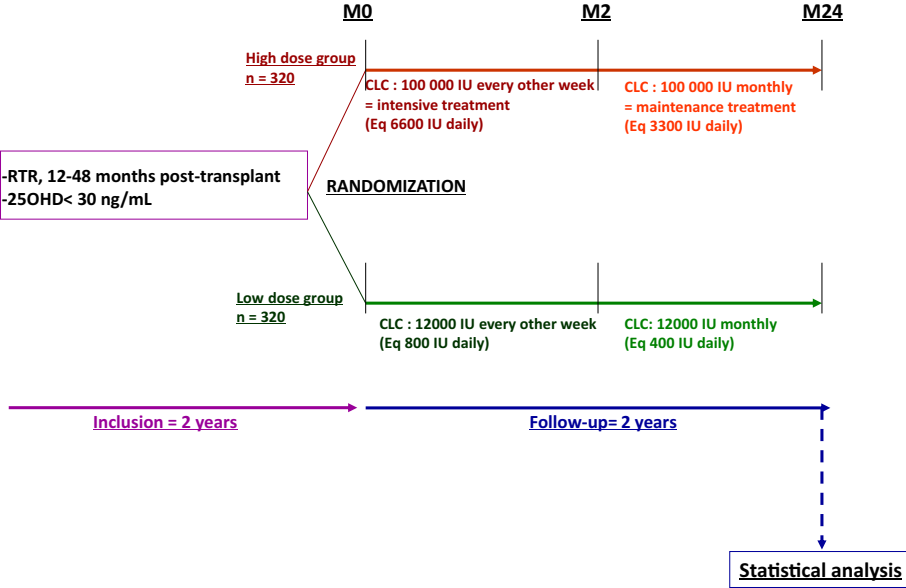

Supplement: Supplementary file 1 — Authors’ original file for figure 1 [file 13063_2014_2292_MOESM1_ESM.pdf]
